# It Is Hard to Read Minds without Words: Cues to Use to Achieve Empathic Accuracy

**DOI:** 10.3390/jintelligence9020027

**Published:** 2021-05-17

**Authors:** Sara D. Hodges, Murat Kezer

**Affiliations:** Department of Psychology, University of Oregon, Eugene, OR 97403-1227, USA; mkezer@uoregon.edu

**Keywords:** empathic accuracy, interpersonal sensitivity, intergroup understanding

## Abstract

When faced with the task of trying to “read” a stranger’s thoughts, what cues can perceivers use? We explore two predictors of empathic accuracy (the ability to accurately infer another person’s thoughts): use of stereotypes about the target’s group, and use of the target’s own words. A sample of 326 White American undergraduate students were asked to infer the dynamic thoughts of Middle Eastern male targets, using Ickes’ (Ickes et al. 1990) empathic accuracy paradigm. We predicted use of stereotypes would reduce empathic accuracy because the stereotypes would be negative and inaccurate. However, more stereotypical inferences about the target’s thoughts actually predicted greater empathic accuracy, a pattern in line with past work on the role of stereotypes in empathic accuracy (Lewis et al. 2012), perhaps because the stereotypes of Middle Easterners (collected from a sample of 60 participants drawn from the same population) were less negative than expected. In addition, perceivers who inferred that the targets were thinking thoughts that more closely matched what the target was saying out loud were more empathically accurate. Despite the fact that words can be used intentionally to obscure what a target is thinking, they appear to be a useful cue to empathic accuracy, even in tricky contexts that cross cultural lines.

## 1. It Is Hard to Read Minds without Words: Cues to Use to Achieve Empathic Accuracy

The ability to accurately read others’ thoughts is a much-desired skill, to the point of being associated with having magical superpowers (Ickes 2008; Marist Poll 2011). However, unlike other talents attributed to superheroes that we mere mortal humans have no chance of achieving (such as telescopic vision or shooting spider webs from our wrists), people actually have some rudimentary ability to infer other people’s thoughts—not very accurately, but with some degree of accuracy greater than chance (Hodges et al. 2015). Furthermore, unlike superpowers, which tend to be bimodal (people either have them or not), empathic accuracy is more like many other human socio-emotional abilities, in that people show variation in performance (e.g., Lewis et al. 2012). In this paper, we focus on two sources of information people can use to construct the contents of a stranger’s mind that do rely not on super powers, but on information readily available to everyday “mind-readers.” The first is the words uttered out loud by the target person whose thoughts are being inferred. The second is stereotypes about a group to which the target belongs. We examine both in a tricky intergroup context that involves understanding across cultural lines.

## 2. Defining Empathic Accuracy

The ability to correctly infer what other people are thinking and feeling, as those thoughts and feelings dynamically change over time, is known as empathic accuracy (Hodges et al. 2015; Ickes and Hodges 2013). In the early 1990s, Ickes and colleagues (Ickes et al. 1990) developed a performance-based method for measuring empathic accuracy that asks targets to report the content of their feelings and thoughts^1^ at a particular moment in time. It then credits perceivers for how closely they can approximate a description of what the target reports is in their head.

Ickes’ paradigm distinguished itself from other measures of interpersonal sensitivity or accuracy in several ways. First, the dynamic element is key. Rather than asking perceivers to decode static facial expression (e.g., like the DANVA; Nowicki and Duke 1994; or the Reading the Mind in Eyes test, e.g., Baron-Cohen et al. 2001) or to guess a target’s overall disposition (e.g., Kolar et al. 1996), Ickes’ empathic accuracy paradigm captures a perceiver’s ability to track *changes* in the thoughts and feelings of the target whom the perceiver is trying to “read.” The perceiver is trying to recreate—or at least piece together—what happens in the “personal movie” running through the target’s head.

This “personal movie” metaphor captures a second important element of Ickes’ empathic accuracy paradigm—that the perceiver’s accuracy attempts include trying to infer cognitive components of the target’s subjective experience, not just the target’s valence and intensity of affect (as other methods have done, e.g., Levenson and Ruef 1992; Zaki et al. 2009). Knowing merely that a person at a bar is feeling positive glosses over a lot of mental content: is it because she is glad to at happy hour after a hard day at work, or is she pleased that the bar patron next to her is flirting with her?

Ickes’ paradigm captures how good (or not so good) humans are at the coveted superpower of knowing the subjective experience of what is going on in someone else’s head. However, using Ickes’ paradigm, we find that, unlike superheroes and science fiction characters who seem to gain instantaneous and full access to another person’s mind, human empathic accuracy attempts may be better described as effortful, piece meal, and incomplete. People gather, retrieve, and integrate a variety of cues when trying to infer others’ thoughts—and some of which may lead to greater accuracy than others. These cues include *generalizations* about what people by and large tend to think and feel in certain contexts—for example, most people feel sad when a close other dies, or feel frustrated when plans are interrupted by events outside their control. These cues can also include information that is specific to an *individual*—for example, knowledge of a friend’s religious beliefs or a co-worker’s feelings about working overtime that may help us infer their thoughts and feelings in ways we could not do for a stranger (see Stinson and Ickes 1992). In this paper, we focus on the utility of two kinds of cues that come with targets, even if they are strangers: what the target is saying out loud, and generalizations about groups to which the target belongs.

## 3. Using What Targets Say

At the top of this list of cues to another person’s thoughts and feelings is what the person is expressing—both verbally and nonverbally via “body language” (e.g., postures, facial expressions, and paralinguistic cues like hesitations or raising one’s voice). One could posit that humans’ success as a species, with our social connections and extensive transmission of shared knowledge, is rooted in part in our uniquely sophisticated use of *verbal* language to say precisely about what we are thinking. However, as we will see in this paper, attending to a target’s verbal cues may be an unsung strategy for achieving greater empathic accuracy.

Nonverbal cues seem to have captivated the general public when it comes to cues for inferring the contents of other people’s minds. This may be related to the fact that nonverbal cues have long been connected to detecting deceit—when people suspect a dishonest target, they may distrust that target’s more controllable verbal output (Lawless DesJardins and Hodges 2015) and look to “leakier” body language (Porter and ten Brinke 2008; Zuckerman et al. 1981). However, despite the allure of relying on body language for deep insight into someone’s true thoughts, researchers studying communication channels have found a robust (and remarkably consistent) primacy for verbal information when it comes to empathic accuracy.

The importance of verbal cues for empathic accuracy was highlighted in a study by Gesn and Ickes (1999) which asked perceiver participants to infer the thoughts and feelings of filmed targets. Perceivers who were deprived of video and heard only the words of the targets did not reliably differ from perceivers who got both audio and video, and both of these groups of perceivers performed significantly better than a third group who saw the video but heard only a filtered audio track that obscured the target’s discernible words. In 2007, Hall and Schmid Mast again found that audio plus video or just audio of a target track produced similarly high levels of empathic accuracy that both significantly exceeded that achieved with just video of a target. In addition, Hall and Schmid Mast ran a condition in which perceivers were asked to guess the target’s thoughts from just a transcript of the interaction. Participants in the transcript condition were deprived not only of many cues, but, in having to read the transcript, were also given a harder cognitive task, and indeed, perceivers receiving just the transcript did worse than perceivers who received audio plus video or just audio. However, perceivers in the transcript condition were more empathically accurate than those who received just video.

Of course, targets may not always say out loud what they are thinking—and not only when they are intending to deceive. For example, target may have already moved on to different thoughts, such as wondering how the perceiver will interpret what the target previously said, being distracted by some activity occurring on the periphery of the conversation, or reviewing mundane plans about what to eat for dinner. Regardless the reason, it is not at all surprising that in the Ickes paradigm, perceivers generally score higher on empathic accuracy when attempting to infer the thoughts of targets who say (out loud) what they are thinking, at the time they are thinking it. This predictor of empathic accuracy is importantly not something about the *perceiver’s* skill, but instead a quality of targets referred to as “transparency” or simply “target difficulty” (e.g., Lewis et al. 2012; Marangoni et al. 1995; Thomas and Maio 2008). This transparency does not necessarily mean honesty, any more than frosted glass on a window means dishonesty. Transparency simply refers to a correspondence between what targets are explicitly sharing and what is in their heads; their reasons for not being transparent may reflect any number of things, including consideration, caution, or cultural norms.

## 4. Using Stereotypes

Even when people are not saying what they are thinking, we can infer their thoughts using cues associated with their group memberships—or in other words, stereotypes (Lewis et al. 2012). The idea that something as non-individuating as stereotypes may help us to accurately infer something as personal as another person’s thoughts is somewhat counterintuitive. It is perhaps further confused by the term “empathic” in empathic accuracy, which suggests a compassionate, or at least benevolent, impetus for accurately inferring others’ thoughts (even though “empathic” accuracy can be used for decidedly unempathic purposes—Hodges and Myers 2007). Acknowledged or intentional use of stereotypes is generally condemned and considered far from “empathic.” Stereotypes play a key role in perpetuating devastating prejudice and discrimination, especially towards minority groups (e.g., Correll et al. 2002; Eberhardt et al. 2006; Spencer et al. 1999). Furthermore, perceiving oneself as stereotyped has a myriad of negative effects (see Steele 2010), and being perceived merely as an interchangeable representative of one’s group, even a positively perceived one, can diminish one’s individual identity and have other negative consequences (Gupta et al. 2011; Wout et al. 2009).

However, stereotypes are a fundamental part of how humans comprehend other people. As is the case with other cognitive templates (such as scripts and schemas) that allow us to absorb wider swathes of information than possible if we processed each stimulus in a bottom-up fashion, stereotypes can provide a quickly-rendered, broad-stroke first draft of our portrait of another person. Stereotypes play an important role largely outside our awareness in how we perceive others and despite the clearly documented negative side of using stereotypes, they can contribute in significant ways to interpersonal accuracy (Jussim et al. 2015). Stereotypes are what guide us to order our meal from a server in a restaurant, rather than a fellow dining patron, or to choose a movie that will likely entertain but not traumatize a seven-year-old who has unexpectedly joined us for movie night.

Consistent with this useful nature of stereotypes, a previous study by Lewis et al. (2012) found that stereotype use improved empathic accuracy. Perceivers were asked to infer the thoughts and feelings of targets who were talking about recently having become mothers for the first time. Those who made more stereotypic inferences about the targets, e.g., inferring thoughts associated with stereotypes about new mothers, such as being sleep-deprived, loving their babies, and perceiving that their lives have changed dramatically, were also more accurate. However, will using stereotypes benefit empathic accuracy when the stereotypes take a darker turn? New mothers as a group hardly fit our “stereotype” of stereotyped groups: they are not a small or stigmatized minority and are generally an admired group. Stereotypes improved empathic accuracy in Lewis et al.’s (2012) study because the stereotypes about new mothers were correct: the new mothers’ thoughts and feelings contained the same stereotypic content that the perceivers associated with the role of being a new mother (see also Hodges et al. 2010). Specifically, stereotypes were helpful for empathic accuracy because the stereotypes were accurate. For this reason, as stereotypes decrease in accuracy, due to bias, prejudice, or ignorance, they should also contribute less to accuracy and to the extent they are still used, actually start to predict *in*accuracy.

Thus, the present study examined use of stereotypes in empathic accuracy in reading targets from a group that is stereotyped in the more usual negative sense associated with the word “stereotype.” In the U.S., and in many countries in Western Europe, there is a negative stereotype for people from what is conversationally referred to as the “Middle East” (but is more thoroughly described as MENA—the Middle East and North Africa^2^). Investigations of American media have found that representations of Arabs and Middle Easterners are one-sided, homogenous, and infused with themes of barbarism and misogyny (Nacos and Torres-Reyna 2007; Shaheen 2003; Sisler 2008) and accompany an alarming rise in hostility against Middle Easterners and Muslims (the predominant religion in the Middle East, although many Muslims are from other regions of the world), both after the 9/11 attacks on the U.S., and under the Trump presidency (e.g., Lichtblau 2016). A qualitative study (Modir and Kia-Keating 2018) found that Middle Eastern students in American colleges reported facing a multitude of discriminatory experiences on and off-campus, including being derogatorily labeled as terrorists by their peers and community members. Individuals on their campus appeared to have little knowledge and awareness of Middle Easterners’ ethnic and cultural backgrounds.

We hypothesized that this lack of knowledge, combined with negative bias, would *reverse* the pattern found in Lewis et al. (2012), such that use of stereotypes would be negatively associated with empathic accuracy. We purposefully chose Middle Eastern men as our target stereotyped group, because Middle Easterners make up a fairly small group in the U.S., meaning that many Americans may not know many—or any—people from the Middle East, allowing misrepresentations in both commercial and social media to flourish in the absence of actual personal interactions. We chose to focus on just *men* from the Middle East, because previous research has found that the stereotypes associated with Middle Eastern men differ from those associated with Middle Eastern women (Ghavami and Peplau 2012).

## 5. The Current Study

In the current study, we took a novel look at two sources of cues that perceivers might—for better or worse—use to infer a stranger’s thoughts: using the target’s words and using stereotypes about the target’s group. Lewis et al.’s (2012) study found that use of stereotypes about a group that is benignly or even positively stereotyped increased empathic accuracy. In contrast, in the current study, although we are still predicting that perceivers will use stereotypes in inferring targets’ thoughts, we predict the *reverse* effect of stereotypes on accuracy because the group stereotype is negatively biased. In addition, we novelly build on two previous studies (Gesn and Ickes 1999; Hall and Schmid Mast 2007) that demonstrated in an all-or-nothing fashion that having access to a target’s verbal cues substantially contributes to perceivers’ empathic accuracy. Here, we introduce a new measure that quantifies *how much* perceivers’ use of targets’ spoken words can be used to predict their empathic accuracy.

**Hypothesis** **1 (H1).** *White perceivers’ use of stereotypes associated with Middle Eastern men to infer the thoughts and feelings of target men from Middle Eastern countries will negatively predict empathic accuracy.*

**Hypothesis** **2 (H2).** *Perceivers’ use of the words spoken aloud by Middle Eastern male targets to infer the men’s thoughts will positively predict empathic accuracy. In order to measure how much perceivers used the content from the targets’ speech to infer the targets’ thoughts, a new rating scale was developed, assessing how closely the content of a perceiver’s inference matched what the target was saying out loud at the same moment that the target had reported a thought or feeling.*

As noted earlier, previous empathic accuracy studies have also found that perceivers are better at inferring targets’ thoughts when the thoughts are high in transparency, i.e., when the targets’ thoughts are rated as being easy rather than difficult to infer based on the information that a target has shared verbally (e.g., Lewis et al. 2012; Marangoni et al. 1995; Thomas and Maio 2008). This study’s third hypothesis is simply that, in line with past work, perceivers’ empathic accuracy will increase as the transparency of the targets’ thoughts increases.

**Hypothesis** **3 (H3).** *The transparency of the Middle Eastern men’s thoughts, or how similar what the targets say aloud is to what the targets report actually thinking, will positively predict empathic accuracy.*

## 6. Moderating Effects of Thought Stereotypicality and Thought Transparency

Hypothesis 1 predicts that, on average, perceivers’ use of stereotypes will negatively predict empathic accuracy, but previous work has found that there is variability in the stereotypicality of people’s thoughts. When Lewis et al. (2012) investigated the effectiveness of perceivers’ use of stereotypes in inferring new mothers’ thoughts, they found that stereotypes’ contribution to accuracy was moderated by how stereotypic targets’ thoughts were: use of stereotypes was especially predictive of empathic accuracy for thoughts high in stereotypicality. Thus, in the current study, we might also expect the effectiveness of using stereotypes to infer others’ thoughts to vary from thought to thought: when the *target’s thoughts* are low on stereotypicality (something we might expect to occur often in the current study, given that we are anticipating that the stereotypes of their group—at least those held by others—are negatively biased), *perceivers’ use* of stereotypes will be an even less effective strategy for accurately inferring others’ thoughts than when the target’s thoughts are higher in stereotypicality.

**Hypothesis** **4 (H4).** *Stereotypicality of targets’ thoughts will interact with perceivers’ use of stereotypes such that when thoughts are low on stereotypicality, perceivers’ use of stereotypes will negatively predict empathic accuracy, but when thoughts are high on stereotypicality, perceivers’ use of stereotypes will positively predict empathic accuracy.*

Finally, because previous research has found that the accuracy of perceivers’ inferences varies depending on the transparency of targets’ thoughts, we predicted that the effectiveness of using targets’ spoken words to infer the targets’ thoughts would be moderated by the transparency of targets’ thoughts.

**Hypothesis** **5 (H5).** *Transparency of the targets’ thoughts will interact with perceivers’ use of the targets’ spoken words such that the perceivers’ use of the targets’ spoken words will only positively predict empathic accuracy when the targets’ thoughts are highly transparent (i.e., when targets are saying out loud what they also report thinking).*

These five hypotheses were pre-registered on the Open Science Framework (https://osf.io/desx8, accessed 11 May 2021). The hypotheses were tested using Ickes’ “standard stimulus paradigm” for measuring empathic accuracy (see Ickes and Hodges 2013), but with multiple “standard” target stimuli. Multiple perceivers watched pre-recorded interviews of targets and were asked to make their best inference about what the targets were thinking or feeling at specific moments in time. We also asked a separate group of subjects from the same subject pool to tell us about the stereotypes associated with Middle Eastern men, so that we could later code our targets’ thoughts and feelings, and our perceivers’ inferences, for stereotypicality. In addition, targets’ thoughts were coded for transparency (how much their reported thought/feeling matched the words they were speaking out loud at the point in the interview when they reported that thought), and perceivers’ inferences were coded for “use of targets’ words” (how much their inferences for a particular thought/feeling matched what the target was saying out loud at that point in the interview). Due to the nested nature of this study’s design, a multilevel model was used to analyze the relationship between this study’s predictors and empathic accuracy and to account for variability among thoughts, targets, and perceivers.

## 7. Method

### Procedure

**Target Interview Phase.** Nine men (*M_age_* = 23.4, *SD* = 4.9) who grew up in the Middle East, including Saudi Arabia, Iraq, Iran, the United Arab Emirates, and the Palestinian Territory, served as targets. They were recruited from local institutions of higher education and the community via word of mouth, flyers, e-mail, and the University of Oregon Psychology and Linguistics Human Subjects Pool. All the targets were currently either graduate or undergraduate students and were compensated with either $20 or 1.5 hours’ worth of participation towards a course assignment for participating in the Psychology and Linguistics Human Subjects Pool.

To create the target stimuli, these men were filmed while answering a set of questions about their beliefs and opinions. The questions, asked by a White female graduate student, covered general topics (i.e., “How would you describe yourself?”) as well as topics more specifically associated with stereotypes regarding Middle Eastern men’s beliefs in American society (i.e., “What differences, if any, have you noticed about the role women have in American society versus in Middle Eastern society?”). A complete list of the interview questions is included in Appendix A. The full interviews lasted approximately 10 min each.

After each interview, in line with Ickes’ empathic accuracy paradigm, the targets watched the recording of their interview and were asked to pause the tape each time they remembered having a thought or feeling during the interview. Each time the recording was paused, the targets were given the prompt, “I was thinking/feeling…” and asked to report (via typing on a computer) the thought or feeling that they remembered having at that moment^3^, as well as the time on the recording at which the thought or feeling occurred. Prior to using the targets’ interviews as stimuli, each recording was “chunked” into clips such that each clip ended at the exact moment that a target reported having a thought or feeling. These clips were used to create the final set of stimuli, which included three to six consecutive clips per interview (the number varied based on the number of thoughts/feelings that the targets reported). Following the interviews, targets completed a demographics questionnaire.

**Empathic Accuracy Assessment Phase.** Shooting for a preregistration goal of 352 useable participants, we recruited 394 undergraduate students from the University of Oregon Psychology and Linguistics Human Subjects Pool to participate as perceivers in the Empathic Accuracy Phase of this study. Perceivers were compensated with partial credit towards a course assignment for taking part in this study. Perceivers first completed the empathic accuracy task and then a questionnaire with demographics and exploratory individual differences measures. Due to computer/experimenter issues, we failed to collect empathic accuracy data for 25 perceivers. In addition, although there is some evidence that some minority members hold the same implicit associations about minority groups as majority members (Assari 2018; Morin 2015), we excluded data from the 38 perceivers who provided any response other than “European American/Caucasian/White” to a demographics question. Finally, we excluded 1 perceiver for not following instructions on the empathic accuracy task and 4 for failing an attention check on the final questionnaire. This left a sample of 326 (*M_age_* = 20.0, *SD* = 3.4; 190 female, 133 male, and 3 who did not provide their gender identification).

To measure empathic accuracy, perceivers each watched clips from four of the target interviews. We randomly assigned perceivers to watch one of nine sets of four interviews that were created by using a pseudo Latin-Square method. At each moment that a clip stopped, the perceivers were asked to report (via typing on a computer) their best inference for what the target in the video was thinking or feeling at that moment in time. Perceivers then completed a demographics questionnaire and for exploratory purposes only, several self-report measures.

**Stereotype Collection Phase.** To find out what stereotypes about Middle Eastern men were familiar to perceivers in the Empathic Accuracy Assessment Phase of this study (described above), we asked a separate group 60 University of Oregon undergraduates about stereotypes associated with Middle Eastern men (*M_age_* = 20.4, *SD* = 2.2; 35 female, 25 male; 11.6% Asian or Asian American, 1.6% Black or African American, 16.6% Latinex/Hispanic/Chicano/Puerto Rican, 5% Middle Eastern or North African, 10% multiracial, 55% White or European American^4^). Participants were compensated with partial credit toward a class research assignment and came from the same pool as perceiver participants used during the Empathic Accuracy Assessment Phase of this study (described above), but students could only participate in one phase or the other of this study. We intentionally collected the stereotypes from a separate group of research subjects so as not to arouse self-presentational concerns about stereotyping among perceiver participants in the Empathic Accuracy Assessment Phase of this study.

The Stereotype Collection Phase of the research was conducted online, with participants receiving the following instructions: “We are interested in understanding what stereotypes are associated with Middle Eastern men. Although you may not endorse these stereotypes yourself, you may still be familiar with what the stereotypes associated with Middle Eastern men are. Using your knowledge of stereotypes that exist about Middle Eastern men, please describe what thoughts you think the stereotypical Middle Eastern man would have in response to being asked each of the following questions.”

Then, participants were presented with each of the questions that the Middle Eastern targets were asked during the Target Interview Phase of this study (see Appendix A) and asked to describe what they thought a stereotypical Middle Eastern man would be thinking if he were asked that question. For example, participants were asked what they thought a stereotypical Middle Eastern man would be thinking if he were asked the question, “How would you describe the country where you grew up?” We emphasized in the instructions that we were interested in the participants’ knowledge of stereotypes that are associated with Middle Eastern men in American society, even if they did not endorse those stereotypes themselves (previous research has found that people have knowledge of stereotypes, even if they do not endorse the stereotypes, e.g., Devine 1989). Participants completed a demographics questionnaire and for exploratory purposes only, several self-report individual difference measures.

The participants’ responses were independently content-analyzed by two White female graduate students who identified common themes shared by the responses. For instance, in response to the question, “What do you think a stereotypical Middle Eastern man would be thinking if he were asked the question, ‘How would you describe the country where you grew up?’” the following responses were grouped together: “Beautiful but war ridden”; “It was war torn and had strict religious laws”; “The country I grew up in is damaged with war and terror. There are bombings and shootings throughout the country by terrorist groups and other countries’ armies.” The common stereotypic theme that had been identified as shared among these responses was that a stereotypical Middle Eastern man would think about the country where he grew up as “violent/war ridden/in conflict.” Stereotypic themes were retained if they were mentioned by at least five participants. Based on this criterion, initially, about 60 stereotype themes emerged; the complete list can be found in Appendix B. Notably, although some of the themes seemed to reflect a negative stereotype of Middle Eastern men (e.g., Middle Eastern men perceive American women as immodest/have too much power/freedom), we were surprised how many of the themes seemed neutral (e.g., Middle Eastern men like spicy food; like to eat/drink/talk with friends) or even positive (e.g., Middle Eastern men would describe themselves as good/kind)—we will return to this point in the discussion.

**Empathic accuracy scores.** Scores from the Empathic Accuracy Assessment Phase were coded and computed using the procedure described in Lewis et al. (2012). Independent undergraduate research assistants compared each of the perceivers’ inferences to the corresponding thought/feeling that the target reported having during their interviews. Each inference by the perceivers was coded by at least three coders. The inferences were rated for accuracy using a 4-point scale, ranging from 0 (the inferred content and the reported thought/feeling content were not the same) to 3 (the inferred content captures the gist of the reported thought/feeling content—all elements of the content were there, and nothing was incorrect). Because the data were collected over multiple academic terms, different teams of undergraduates coded empathic accuracy; interrater reliability for these teams was acceptable (ranging from Cronbach’s α = .64 to .79). Empathic accuracy ratings were averaged across coders, and then, as is customarily done with Ickes’ paradigm, they were divided by the maximum possible accuracy score (3.0) and converted to a 0 to 1.0 scale.

**Perceivers’ use of the targets’ spoken words.** The perceivers’ use of the targets’ spoken words to infer the targets’ thoughts/feelings was coded by a different team of undergraduate research assistants. Each inference by the perceivers was coded by at least four coders. In a procedure similar to that followed by the perceivers themselves, the coders watched consecutive clips from each of the targets’ interviews, each of which was stopped at the same moment they had been stopped for the perceivers who were asked to infer what the target was thinking at the end of each clip. After each clip ended, coders rated how similar each of the perceivers’ inferences about the target’s thought/feeling at that moment was to what the target had said out loud in the interview thus far. The coders rated each inference using a 4-point scale, ranging from 0 (the inference did not match the targets’ verbalized responses at all) to 3 (the inference matched the targets’ verbalized responses). The interrater reliability was acceptable (ranging from Cronbach’s α = .72 to .76). Use of spoken word ratings were averaged across coders.

**Transparency of targets’ thoughts.** The transparency of the targets’ thoughts (how much the target’s words spoken out loud matched what the target reported thinking or feeling) was rated by the same teams of undergraduate coders who assessed the perceivers’ use of the targets’ spoken words. Transparency coding was adapted from previous research (Marangoni et al. 1995): coders read the target’s reported thought/feeling at the end of each video clip and then rated how closely it matched what the target had said out loud in the interview thus far. The coders used a 4-point scale from 0 (the reported thought/feeling does not match the targets’ out loud verbalized responses at all) to 3 (the reported thought/feeling matches the targets’ verbalized responses). An example of a thought high in transparency in the present study would be the target who reported “I thought about a word in English,” just after saying said out loud, “I am trying to think of the word in English, um…um…” In contrast, a thought that was low in transparency was the target who reported, “I was thinking about an idea I had recently which is don’t follow yourself,” while saying out loud “Actually when anyone asks me to describe myself, I don’t like to describe myself—nothing much.” The interrater reliability was acceptable (Cronbach’s α = .84). Ratings were averaged across coders.

**Perceivers’ Use of Stereotypes.** The perceivers’ use of stereotypes to infer the targets’ thoughts was assessed using four coders. Each inference was coded by at least two coders. Two of the coders had previously coded the inferences for the perceivers’ use of the targets’ spoken words, and the other two coders had previously coded the inferences for empathic accuracy. This resulted in the four coders having already seen the inferences once when rating the inferences for the perceivers’ use of stereotypes; however, we did not think this earlier exposure would systematically bias the stereotypicality coding in any way.

To rate how much perceivers’ inferences contained stereotypes associated with Middle Eastern men, the coders compared each inference to the stereotypic themes collected during the Stereotype Collection Phase of this study (described earlier) and rated how closely an inference matched any of the stereotype themes. Ratings were made on an asymmetrical 4-point scale: a rating of 4 was assigned to an inference that closely matched one of the stereotype themes; a rating of 3 was assigned to an inference that somewhat matched one of the stereotype themes; a rating of 2 was assigned if the inference did not mention any stereotypic themes, and a rating of 1 was assigned if an inference explicitly countered one of the stereotype themes. (We did not use a 5-point symmetrical scale, because in developing the coding scheme, it became clear that it was very hard to differentiate between an inference that was somewhat counter-stereotypic and one that was very counter-stereotypic.) Thus, higher ratings indicated greater stereotypic content. The interrater reliability was good (ranging from Cronbach’s α = .94 to .96). To create a composite score for each inference, the ratings were averaged across coders. 

**Stereotypicality of targets’ thoughts.** Stereotypicality ratings of the targets’ thoughts were completed in the same session as the stereotypicality ratings of perceivers’ inferences and with the same four coders. Each of the target’s thoughts/feelings was coded by at least two coders. The coders rated the target’s stereotypicality using the same 4-point scale as was used to rate the stereotypicality of the perceivers’ inferences (see above). The interrater reliability was good (Cronbach’s α = .81). To create a composite stereotypicality score for each thought, the ratings were averaged across coders.

## 8. Results

All analyses were performed in R using the *lme4* package (Bates et al. 2018). Data and R code are available at https://osf.io/582ef/.

We fit a multilevel model in which thoughts were nested within targets, which were crossed with perceivers. The model was tested using restricted maximum-likelihood estimation and grand mean-centered predictor variables. The model included random effects of the intercept for thoughts, targets, and perceivers, and the other effects were treated as fixed^5^ due to the small number of observations at Level 1 (i.e., number of thoughts per target). This multilevel model is represented by the following equations where we follow the Gelman and Hill (2006) notation extracted by *equatiomatic* package (Anderson et al. 2021):

$$\begin{array}{c} {\mathrm{EmpathicAccuracy}}_{i}\sim N\left(\alpha_{j\left[i\right],k\left[i\right],l\left[i\right]}+\beta_{1}\left({\mathrm{UseOfTargets}}^{\prime }\mathrm{Words}\right)+\beta_{2}\left(\mathrm{UseOfStreotypes}\right),\sigma^{2}\right)\\ \alpha_{j}\sim N\left(\gamma_{0}^{\alpha }+\gamma_{1}^{\alpha }\left(Gender_{1}\right),\sigma_{\alpha_{j}}^{2}\right),\mathrm{for}\;\mathrm{Participant}\;j=1,\ldots ,J\\ \;\;\;\;\;\;\;\;\;\;\;\;\;\;\;\;\;\;\;\;\;\;\begin{array}{cc} \alpha_{k}\sim N(\gamma_{0}^{\alpha }+\gamma_{1}^{\alpha } & \left(\mathrm{ThoughtTransparency}\right)+\gamma_{2}^{\alpha }\left(\mathrm{ThoughtStereotypicality}\right)\\ & +\gamma_{3}^{\alpha }\left({\mathrm{UseOfTargets}}^{\prime }\mathrm{Words}\times \mathrm{ThoughtTransparency}\right)\\ & +\gamma_{4}^{\alpha }\left(\mathrm{UseOfStreotyp}\times \mathrm{ThoughtStereotypicality}\right),\sigma_{\alpha_{k}}^{2}),\\ & \mathrm{for}\;\mathrm{Thought}:\mathrm{Target}\;k=1,\ldots ,K \end{array}\\ \alpha_{l}\sim N\left({µ}_{\alpha_{l}},\sigma_{\alpha_{l}}^{2}\right),\mathrm{for}\;\mathrm{Target}\;l=1,\ldots ,L\;\;\;\;\;\;\;\;\;\;\;\;\;\;\;\;\;\;\;\;\;\;\;\;\;\;\;\;\;\;\;\;\;\;\;\;\; \end{array}$$

where *i* represents the inference, *j* represents the participant, *k* represents the thought, and *l* represents target. Because variability in empathic accuracy scores can be attributed to variations among thoughts, targets, and perceivers, the model included random intercepts for thoughts, targets, and perceivers.

An inspection of the residuals of the outcome variable revealed that its distribution was positively skewed, violating the assumption of normality of residuals. Therefore, we transformed the empathic accuracy scores using Box–Cox transformation as described in (Cohen et al. 2002) with *MASS* package in R (Ripley et al. 2013). In reporting the multilevel analyses, we report only the results using the transformed outcome variable. (See OSF page for the results using non-transformed empathic accuracy scores.)

The descriptive statistics for non-transformed empathic accuracy and predictor variables are reported in Table 1 below.

The descriptive statistics indicate that, on average, perceivers were not very accurate at inferring the targets’ thoughts (*M* = .09, on a 0 to 1.0 scale) and empathic accuracy was lower than found in previous studies (e.g., Lewis et al. 2012). Turning to stereotypic content, given the scale used to measure stereotypicality, where “2” indicated neither stereotypic nor counter-stereotypic, both perceivers’ inferences (*M* = 2.15) and targets’ thoughts (*M* = 2.2) were on average only slightly, but not highly, infused with the themes gathered during the Stereotype Collection Phase of this study. Both perceivers’ inferences (*M* = .55) and targets’ thoughts (*M* = .73) tended to be low on the degree to which they matched the targets’ spoken words, where the maximum rating was 3.0.

Intraclass correlation coefficients (ICCs) were calculated to analyze descriptively the amount of variability in empathic accuracy scores that could be explained by the random effects that were included in the model. The ICCs indicate that approximately 11% of the variance in model intercepts was explained by variations among thoughts relative to the targets to which those thoughts belong (Variance Estimate = .00029), 8% by variations among targets (Variance Estimate = .00019), and 11% by variations among perceivers (Variance Estimate = .00023).

**Figure 1:**
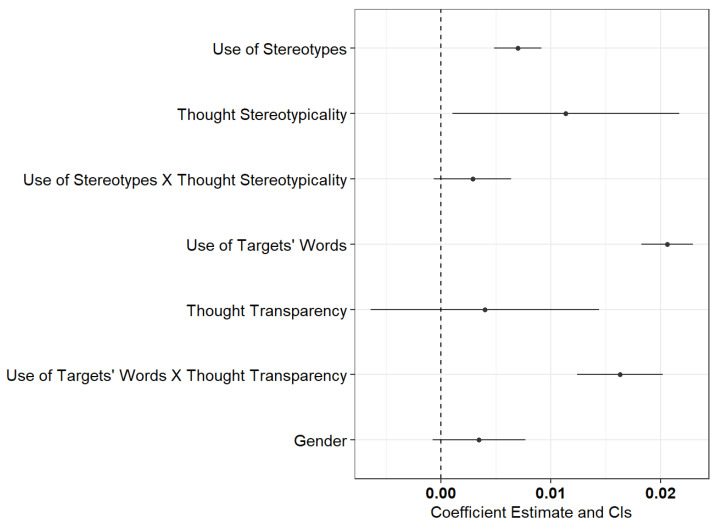
Parameter estimates for the fixed effects [95% CI].

The results for the fixed parameters resulting from the multilevel model analysis are shown in Table 2 below, and Figure 1 provides a visual representation of the fixed effects with confidence intervals.

Our first hypothesis was that White American perceivers’ use of stereotypes about Middle Eastern men when inferring the thoughts of Middle Eastern male targets would negatively predict empathic accuracy. Contrary to our expectation, perceivers’ use of stereotypes *positively* predicted empathic accuracy, *b* = .01, *SE* = .001, *p* < .001. These results did not support our study’s first hypothesis. Instead, they were the same as—not the reverse of—Lewis et al.’s (2012) findings, which also found that more stereotypic inferences were associated with greater empathic accuracy. We had hypothesized the opposite pattern for the current study, because our targets were members of a group about which there are negative and inaccurate stereotypes, unlike the new mother target group used in Lewis et al.’s study—a point we will return to in the discussion. In addition to the main effect of stereotypical inferences (in the opposite direction predicted), we also found a non-predicted main effect of thought stereotypicality, *b* = .01, *SE* = .01, *p* = .03—perceivers were more empathically accurate when targets’ thoughts were more stereotypic.

Our second hypothesis, that perceivers’ use of the words spoken out loud by the Middle Eastern male targets to infer the targets’ thoughts would positively predict empathic accuracy, was supported. The significant main effect of perceivers’ use of the targets’ spoken words on empathic accuracy, *b* = .02, *SE* = .001, *p* < .001, indicates that as perceivers’ use of targets’ spoken words increased, empathic accuracy also increased.

Our third hypothesis was simply to replicate previous unsurprising findings that the transparency of the targets’ thoughts—or in other words, how similar what the targets said aloud was to the thoughts that they reported having—would positively predict empathic accuracy. However, this hypothesis was not supported: thought transparency did not significantly predict empathic accuracy, *b* = .004, *SE* = .01, *p* = .45.

Our fourth hypothesis was that there would be a significant interaction between the perceivers’ use of stereotypes and the stereotypicality of targets’ thoughts such that, when targets’ thoughts were low on stereotypicality, perceivers’ use of stereotypes would negatively predict empathic accuracy, and when targets’ thoughts were high on stereotypicality, perceivers’ use of stereotypes would positively predict empathic accuracy. However, contrary to both our hypothesis and past results by Lewis et al. (2012), there was no significant interaction between perceivers’ use of stereotypes and the stereotypicality of targets’ thoughts on empathic accuracy, *b* = .003, *SE* = .002, *p* = .12.

**Figure 2:**
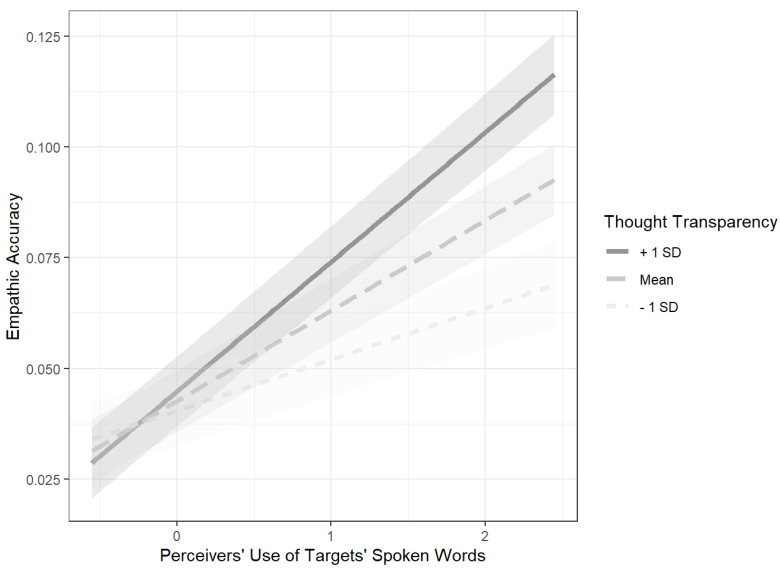
A simple slopes analysis of the interaction between perceivers’ use of the targets’ spoken words and targets’ thought transparency.

Our fifth hypothesis was that there would be a significant interaction between the perceivers’ use of the targets’ spoken words and the transparency of the targets’ thoughts, such that when targets’ thoughts were high on transparency, the perceivers’ use of the targets’ spoken words would positively predict empathic accuracy, and when targets’ thoughts were low on transparency, the perceivers’ use of the targets’ spoken words would not predict empathic accuracy. This hypothesis was confirmed, *b* = .02, *SE* = .002, *p* < .001. In order to unpack this significant interaction effect, a simple slopes analysis was conducted using the *jtools* package in R (Long 2017). The effect of perceivers’ use of the targets’ spoken words on empathic accuracy at +/−1 SD on thought transparency was analyzed and is shown in Figure 2 below.

Consistent with the fifth hypothesis, the results of the simple slopes analysis indicated that when targets’ thoughts were high on transparency (+1 SD), perceivers’ use of the targets’ words significantly, positively predicted empathic accuracy, *b* = .03, *p* < .001. However, contrary to our expectation, when targets’ thoughts were low on transparency (−1 SD), perceivers’ use of the targets’ words was still a significant predictor of empathic accuracy, *b* = .01, *p* < .001.

Because a female advantage for empathic accuracy has been found in some previous studies but not others (e.g., Klein and Hodges 2001; see Hodges et al. 2011 for a review), gender was included as a covariate in the current analysis. We found no effect of gender on empathic accuracy, *b* = .003, *SE* = .002, *p* = .11.^6^

## 9. Discussion

When faced with trying to read the mind of an unfamiliar person—someone who is from a different cultural background to boot—what are the sources of information perceivers can use? Our investigation produced some clear answers in line with our predictions, but also some outcomes that we were not expecting that merit some consideration. Starting with the latter first, our results for the role of stereotypes in empathic accuracy did not support either of our predictions—in fact, they even ran *counter* to one of our predictions, but they did so in a way that was actually consistent with previous results. We had predicted that perceivers’ use of stereotypes to infer Middle Eastern men’s thoughts would hurt empathic accuracy, in contrast to Lewis et al.’s (2012) results that found that use of stereotypes to infer new mothers’ thoughts helped empathic accuracy. Our hypothesis hinged on the fact that, unlike the fairly accurate stereotypes held about new mothers, stereotypes held by White Americans about Middle Eastern men contain negative and incorrect information (e.g., Modir and Kia-Keating 2018; Nacos and Torres-Reyna 2007). However, contrary to our hypothesis, our results showed the *same* pattern as Lewis et al.’s results: White perceivers’ inferences about the Middle Eastern men’s thoughts that were higher in stereotypic content led to greater empathic accuracy.

Our primary guess for our contrary results is that we seem to have overestimated the negativity of the stereotypes that our participant pool associated with Middle Eastern men—or perhaps that our pool would *admit* to associating with Middle Eastern men, maybe due to self-presentational concerns. In addition, we were focused on stereotypes that our perceivers might have about what Middle Eastern men would be thinking about during our interview questions. These questions may have been perceived as unlikely to trigger thoughts in anyone that were related to the most negative aspects of some Middle Eastern male stereotypes.

Our first clue to the relatively mild negativity of the stereotypes was during the Stereotype Collection Phase of this study, when participants drawn from the same population as our perceivers (American university students at a middle-sized public university) provided us with stereotypes of Middle Eastern men. We definitely got some negative content (especially around Middle Eastern men’s attitudes towards women’s roles, and about Middle Easterners’ countries of origin being viewed as poor and war-torn). However, the most virulent characteristics sometimes associated with Middle Easterners in the U.S. media (e.g., “terrorist” or “violent”) did not emerge. Not only did the stereotypes lack extreme negativity, but many of the stereotypic themes we collected were positive (e.g., hard-working; smart). Other themes were more ambiguous in valence—perhaps not explicitly negative, but possibly reflecting caricatures of the ethnic group (e.g., that Middle Eastern men like to eat rice, which could also be seen as related to the derogatory slur of calling Asians and South Asians “rice eaters”).

As additional evidence that the stereotypes were not particularly negative, we ran a quick post-hoc survey among a group of eight naïve undergraduate research assistants who were not involved with collecting or coding the original study’s data. We asked them to categorize the stereotype themes as positive, negative, or neutral—characterizations they independently agreed upon to a large degree. The most common characterization was “neutral” (25 themes); the number of negatively-characterized themes (18) was only slightly higher than the number of positively-characterized themes (16). Two of the themes fell right in the middle between negative and neutral.

A complex question we would like to pursue in future work is whether stereotype valence affects accuracy. In part, when surveying our later team of research assistants about the valence of the original themes, an interesting issue that arose was that the valence of a theme could be dependent on the group in which it was found: White Americans might consider “religious” a positive trait in a White American target, but “religious” might be regarded as a negative trait in a Muslim man from the Middle East. Furthermore, a stereotypic theme seen as neutral in isolation (“likes hummus”) could, depending on the rest of context in which it was embedded, easily take on the more negative cast of a slur (e.g., see our earlier discussion of derogatory use of the term “rice eaters”). This is especially true given that in everyday contexts, recognizing someone’s use of any stereotypes about a racial or ethnic group is generally associated with that person holding a negative judgment about the group. Many Americans—especially American college students—are wary of publicly expressing anything that might be considered racially or ethnically prejudiced, which can make it hard to study the effects of stereotypes (something found repeatedly, for example, when trying to study anti-Black prejudice in the U.S., e.g., Dovidio et al. 2017; Plant and Devine 2001; Sommers and Norton 2006). We think it may be difficult to get college students, who are generally keen on avoiding racism, to endorse any stereotypes of Middle Eastern men as “positive.” That is, regardless if a group is stereotyped as “smart” or “strong,” we have found that first and foremost in many people’s minds is that “you shouldn’t stereotype”; not that “being stereotyped as strong is more positive than being stereotyped as stupid.” To tackle these thorny issues in an investigation of stereotype valence and empathic accuracy that is more conclusive than the post-hoc survey we ran will require additional—and sensitively constructed—research.

Many of the stereotypic themes that emerged during our Stereotype Collection Phase also did not seem particularly specific to Middle Eastern men. Again, turning to our post-hoc survey of eight undergraduate research assistants, only 9 of the 61 themes were characterized as being “uniquely or distinctly” associated with Middle Eastern men (e.g., “favorite food is shawarma”; “member of Muslim religious group”). The remaining themes were about equally split between “somewhat uniquely or distinctly associated” with Middle Eastern men (e.g., “Describes the country he grew up in as having strict rules/traditional”) and “not uniquely or distinctly associated” with Middle Eastern men (e.g., “Best memories from childhood are playing with friends/playing sports”). Prompted by a reviewer’s comment, we asked our group of research assistants about whether the themes were associated with the more general group “men.” Twenty of the 61 themes were seen as “highly associated” (e.g., “describes himself as family man”) or “somewhat associated” (e.g., “best memories from childhood are family time”) with “men”—that is, not just Middle Eastern men. Our small survey (albeit small and post-hoc) thus also provides some support for the idea that, when trying to construct the contents of a specific person’s mind, people may rely on both broader generalizations about people, as well as information that is specific to members of a certain group.

In sum, even though we selected a group about which negative stereotypes can be quite easily found in commercial and social media, the majority of stereotype themes common among our perceiver population were either neutral or positive, and many of them were generalizations that also applied to a broader group of people than just Middle Eastern men. Thus, one assumed tenet about the detrimental effects of stereotypic inferences on empathic accuracy in the current study—that using stereotypes about Middle Eastern men would lead to inaccuracy because they were negatively biased—was at least partially missing.

Lewis et al. (2012) found that using stereotypes of new mothers improved accuracy at least in part because the stereotypes were accurate. Examining the mean stereotypicality for targets’ thoughts and perceivers’ inferences in Table 1 suggests that the stereotypes of Middle Eastern men were not “all” wrong. Both means are just slightly over 2—the point on the stereotypicality scale indicating neither stereotypical nor counter-stereotypical content. Perceivers’ inferences—*and* targets’ actual thoughts—were roughly equal, and equally noncommittal, in terms of containing stereotype themes. Given that the stereotypes were somewhat accurate, it is thus less surprising that the results for stereotypicality in the present study turned out more like Lewis et al.’s results than we had predicted.

Our results also unexpectedly found a main effect of *thought* stereotypicality—perceivers were more empathically accurate when they were inferring thoughts that were stereotypic. Our best guess here is that thoughts that were more stereotypic were generally easier to infer because they were simpler and more straightforward (e.g., positive attitudes such as liking certain foods or soccer; valuing family or religion). The interview questions that we asked of our Middle Eastern male targets (the same questions we presented to participants in our Stereotype Collection Phase) made it more likely that we would get answers from the targets that were stereotype *relevant*—whether those answers were stereotype-consistent or counter-stereotypic. That is, we asked the targets about things such as religious conflict, perceptions of women’s roles, and favorite foods; not about non-stereotype-relevant topics such as how the targets were doing in their academic major or whether they thought they looked more like their mother’s or father’s side of the family. To be sure, in response to our stereotype-relevant questions, we definitely got target responses that were counter-stereotypic. However, the stereotype-relevant questions may also have pulled for a number of straightforward, stereotype-consistent thoughts that were easy for our perceivers to infer (e.g., not all Middle Eastern men like falafel, but if a target mentioned longing for it, our perceivers likely inferred their thoughts about it correctly).

In their study, Lewis et al. also found an interaction of stereotypicality of perceivers’ inferences and stereotypicality of targets’ thoughts—the greatest empathic accuracy occurred when perceivers made highly stereotypic inferences about targets’ highly stereotypic thoughts, i.e., guessing the new mothers’ thoughts were stereotypic only led to empathic accuracy when the women really were thinking stereotypic thoughts. We did not replicate this interaction in the current study, but we suspect this may be due to differences in the two studies. In the current study, our analysis that produced no inference stereotypicality x thought stereotypicality interaction was slightly different from Lewis et al.’s, as the current study included two additional parameters not included in Lewis et al.’s analyses—how much perceivers used targets’ words, and the interaction of this word-use variable with thought transparency. When we re-ran our analyses and left out these two terms, using just the same parameters as in Lewis et al.’s analyses, we replicated the effect Lewis et al. found, with a similar (and significant) interaction between inference stereotypicality and thought stereotypicality (*B* = .01, *p* = .013): the greatest empathic accuracy was seen when perceivers made stereotypic inferences about stereotypic thoughts. These “word-use” parameters (discussed below) may have shared some variance with our parameters related to stereotypicality, so that this interaction was not seen when they were also in the model.

Additionally, our system for coding of stereotypes was more elaborate than Lewis et al.’s (2012) work. In the Lewis et al. paper, coders simply had to read an inference and rate it for stereotypicality, using solely their own judgments about what was stereotypical. In contrast, given that some of stereotype themes about Middle Eastern men are negative, and also given that we were asking earnest and open-minded research assistants to make ratings related to a deeply unpopular activity (i.e., stereotyping of a discriminated group), we gave them an explicit list of the 60 or so stereotype themes to look for when making their ratings of stereotypicality, rather than having them simply consult their own subjective understanding of the stereotypes.

The stereotypes used in the Lewis et al. study may have covered a much shorter list of more commonly agreed-upon themes (whereas to earn a spot on our Middle Eastern stereotypes list, only 5 out of 60 participants had to mention it in our Stereotype Collection Phase of this study). Thus, in Lewis et al.’s study of new mother targets, if a perceiver made a highly stereotypic inference, it was more likely to contain the *same* stereotype that rendered a target’s thought high in stereotypicality, providing a higher probability of matching content (and higher empathic accuracy) as well. In contrast, in the current study, with our long list of themes about stereotypical Middle Eastern men collected from another group of participants, simply matching in terms of levels of stereotypicality was no guarantee that perceivers were inferring the same content that targets were thinking.

Finally, as noted earlier, perceivers’ overall empathic accuracy scores in the current study were quite low. Although the intercept in our multilevel regression equation is positive and significant, indicating *some* accuracy, perceivers were pretty lousy at guessing the Middle Eastern men’s thoughts. This may have been due to a variety of reasons—lack of familiarity with these foreign targets’ experiences, difficulty comprehending their sometimes-accented English, unease about being asked to interact (even asynchronously) with a member of a discriminated-against group (e.g., Gluszek and Dovidio 2010; Holoien et al. 2015; Vorauer and Sasaki 2009)—and quite possibly attempts to avoid doing anything that might be seen as assuming stereotypes. When listening to these strangers from a distant land, stereotypic content might have been one of the few things our American perceivers brought to mind, but if they recognized the content as such, they may have dismissed it in order to avoid being prejudiced. In data from an unpublished project (Lewis et al. 2018) that used the same targets (i.e., new mothers) as Lewis et al. (2012), there was no main effect of explicitly instructing people *not* to use their stereotypes to infer the targets’ thoughts (i.e., these instructions did not hurt their accuracy relative to a control condition). However, instructions did interact with inference stereotypicality: participants who were instructed not to use their stereotypes when inferring the new mother targets’ thoughts, and who followed these instructions by actually making less stereotypic inferences, were also the least accurate.

Does this mean—now with *two* studies finding that more stereotypic inferences lead to greater empathic accuracy—that stereotypes should be promoted as a strategy for increasing empathic accuracy? We still say “no.” There is a difference between people’s judgments reflecting the use of stereotypes and people intentionally using stereotypes, and the current study examines the former, not the latter. In the same unpublished project by Lewis et al. (2018) that is discussed above, another condition in which people were explicitly instructed to *use* their stereotypes to infer the targets’ thoughts also did not improve their accuracy relative to the control condition. The beneficial effect on empathic accuracy of making stereotypic inferences may function more passively, or even spuriously. Trying to guess another person’s thoughts—particularly if that person is a total stranger (e.g., see Stinson and Ickes 1992)—is just a difficult task, and *any* knowledge about the person or context, on average, may help relative to having *no* knowledge. When perceivers’ inferences contain stereotypic content, it may simply reflect that they know *something* about the target, the target’s group, or the target’s situation. This minimal knowledge may have been what helped empathic accuracy in the current study, which, it must be remembered, yielded low levels of empathic accuracy overall, suggesting our perceivers found few helpful cues to go on. Intentionally using stereotypes to infer other people’s thoughts seems unwise in terms of achieving empathic accuracy, as those stereotypic inferences may be indiscriminately paired with non-stereotypic thoughts.

However, the most important reason that we recommend against intentional application of stereotypes as a possible route to increase empathic accuracy, particularly in intergroup contexts such as this one, is simply that choosing to use stereotypes—particularly negative ones—can perpetuate and endorse prejudice against members of minority and stigmatized groups, make them feel undervalued as individuals, and lead to destructive and painful outcomes. If our suspicion that stereotypic inferences in the current study improved empathic accuracy because stereotypes were some of the *only* information our perceivers had about people from the Middle East, then our recommendation to improve empathic accuracy in this context would *not* be to use stereotypes, but instead for more Americans to become better educated about Middle Eastern history, culture, and current events, and become friends with some Middle Easterners (see Stinson and Ickes 1992)!

What else do we recommend, while we are doling out empathic accuracy advice? We now turn our attention to a compelling route to empathic accuracy, newly empirically measured in this study and reinforcing a lesson most of us have been taught since early childhood about the importance of paying attention to what others are saying. Perceivers in our study who took targets at their word (in a more literal sense than usual!) were more empathically accurate. This was the case despite the lack of ease associated with communication across different cultural and ethnic groups (e.g., Duronto et al. 2005; Holoien et al. 2015; Vorauer and Sakamoto 2006) and despite any general misgivings about whether people’s words reflect what they are actually thinking for a variety of reasons covered in the introduction.

The idea that we should listen to what people are saying to know what they are thinking may sound obvious in hindsight—and we suspect that may in part be because this idea resonates with the many admonitions over a lifetime that we have heard about how important listening to what others say is in order to understand them. However, despite this “obviousness,” as far as we know, no one else has ever measured just how important a cue targets’ spoken words are for predicting empathic accuracy. And quite possibly, had we found that use of targets’ words did *not* predict empathic accuracy, that finding would similarly be seen as “obvious”—knowing how wary people can be of trusting others’ words and how Lawless DesJardins and Hodges (2015) found that even just the suggestion that people might not be saying what they are thinking can significantly hurt empathic accuracy for some perceivers. (And, anecdotally, in a quarter century of conversations about empathic accuracy with both researchers and lay people, the first author of this paper has been repeatedly asked about the importance of decoding “body language” as a predictor of empathic accuracy, and never, until starting this project, about the importance of attending to verbal language.)

Interestingly, another variable connecting what targets are actually saying with empathic accuracy—transparency, or how closely the target’s words match what the target reports thinking—was unexpectedly found to be *unrelated* to empathic accuracy in the current study. Often transparency plays a supporting, not starring, role in empathic accuracy studies: it is used as a control variable in order to highlight the contributions of other more focal variables in a study (e.g., see Gesn and Ickes 1999; Marangoni et al. 1995), but it frequently makes a statistically significant contribution. Given that Lewis et al. (2012) found that transparency was a significant predictor in a previous investigation of stereotype usage and empathic accuracy, we expected to find this same result in the present data, but failed to do so. We can speculate that perhaps transparency had a restricted range in the current study, where our Middle Eastern targets were likely cautious about how forthcoming they were about topics that these men may have discovered were touchy or problematic in other conversations with Americans. Talking about one’s experience coming to the U.S. as a Middle Eastern man in a psychology study might be a lot more fraught than being an American woman who is asked to talk about her new baby.

However, thought transparency *did* interact with perceivers’ use of the targets’ words, as was predicted by a hypothesis novel to this study. Although transparent thoughts did not provide an across-the-board boost to empathic accuracy, Figure 2 shows how they amplified the effectiveness of using the strategy of inferring that targets were thinking what they said out loud. In terms of interpersonal intelligence, this interaction is analogous to how good students receive higher overall scores on tests, but do particularly well on easy items. Notably, even when the targets’ words were less reflective of what targets were actually thinking (i.e., less transparent), using those words boosted perceivers’ accuracy.

We had thought attention to targets’ words could potentially backfire in terms of empathic accuracy for low transparency thoughts—that a consistent and unwavering strategy of using a target’s spoken words to infer his thoughts could reduce accuracy for those thoughts when the target was largely *not* saying what he was thinking. However, relying on the target’s words seems to be a solid strategy, even in circumstances when a lot of what targets are thinking gets edited out of what they say out loud, as was the case in the present study where the mean “transparency” score was only .73 (on a 0 to 3 scale). Amid a literature that has revealed surprisingly few correlates of empathic accuracy when using Ickes’ dynamic, content-driven paradigm (see Hodges et al. 2015), the current study’s novel operationalization, measurement, and findings regarding this construct make an important contribution by pointing out a new path to empathic accuracy, even if not all our predictions about perceivers’ use of the targets’ words were exactly supported.

Coding the extent to which inferences matched the words being spoken out loud by the target has not been measured in empathic accuracy research prior to this study, to our knowledge. And notably, we found support for our hypothesis that perceivers’ use of targets’ words would facilitate the already daunting task of empathic accuracy with a stranger, in the extra daunting context of intergroup communication. Of course, it is possible that attending to the target’s words is relatively more effective as a strategy for achieving empathic accuracy *specifically when* the target is a stranger from another country, when perceivers have few other cues or sources of information to rely on. Future empathic accuracy research should explore the effectiveness of using a target’s words with targets of different stripes and in different contexts. If the same pattern of results emerges, then the next research direction would be to instruct people to attend to others’ words, as a possible intervention to increase empathic accuracy.

The current research is not without its limitations. For practical reasons, we used just nine Middle Eastern male targets in this study—a number that can in no way fully represent the millions who make up the group “Middle Eastern men.” Additionally, all of our targets were students who had elected to study in the United States. For this reason, they might not only have been less “stereotypically Middle Eastern,” but they may have also been *perceived* as less stereotypically Middle Eastern. Similarly, our perceiver sample—drawn from a university student population—limits generalization of our findings. We suspect that, among other things, a less educated and less socially conscious population may have produced and used more negative stereotypes. The pattern of results that we found may be specific to our particular group of targets, our American student sample, and/or the interaction of the two—suggesting another direction for future research.

In summary, after dissecting the “superpower” of empathic accuracy, it looks a bit more like an everyday human socio-emotional ability. Granted, it is an ability that is hard to excel at, particularly if the person whose thoughts we are trying to infer is the member of another group—*and* a group against whom there is some prejudice—as was the case here. When we probed the predictors of empathic accuracy, we found that using stereotypes—even those for a group whose stereotype contains some negative elements—led to greater empathic accuracy. We had anticipated the opposite result, but we also had anticipated that the content of the stereotypes about Middle Eastern men would be more negative and simpler. Instead of reflecting highly negative images of Middle Eastern men found in some media, the content was more representative of a much less provocative definition of stereotypes—as generalizations about members of a group. And our parting wisdom for supercharging one’s empathic accuracy powers when heroic feats of interpersonal understanding are required? Listen to others’ words in order to read their minds.

## Figures and Tables

**Table 1:** Descriptive Statistics.

Variable	*M* ^a^	*SD*
Empathic Accuracy	.09	.13
Perceivers’ Use of Stereotypes	2.15	.58
Thought Stereotypicality	2.20	.55
Perceivers’ Use of Targets’ Spoken Words	.55	.60
Thought Transparency	.73	.55

^a^ Grand mean.

**Table 2:** Parameter Estimates for the Fixed Effects.

Parameter	*B*	*SE*	*t*	*p*
Intercept	.04	.01	7.83	**<.001**
Perceivers’ Use of Stereotypes	.01	.001	6.33	**<.001**
Thought Stereotypicality	.01	.01	2.16	**.03**
Use of Stereotypes × Thought Stereotypicality	.003	.002	1.55	.12
Perceivers’ Use of Targets’ Words	.02	.001	17.37	**<.001**
Thought Transparency	.004	.01	.76	.45
Use of Targets’ Words × Thought Transparency	.02	.002	8.18	**<.001**
Gender (−1 = Female, 1 = Male)	.003	.002	1.61	.11

**Bold** above indicates significance at *p* < .01.

## Data Availability

The data presented in this study are openly available at https://doi.org/10.17605/OSF.IO/582EF.

## Notes

1 Targets are asked to report what they were thinking or feeling in Ickes’ paradigm and references to targets’ “thinking” and “thoughts” throughout this paper should be considered inclusive of targets’ reported thoughts *and* feelings; no distinction is made between the two in data collection or analyses.

2 Past U.S. Census surveys, including the 2020 census, categorize individuals with Middle Eastern backgrounds as racially White (Wiltz 2014). However, in this paper, we use ‘White’ to refer to individuals of Caucasian, largely European backgrounds (the current ethnic majority group in the U.S.), and “Middle Eastern” to refer to individuals with ancestry in Arab or non-Arab MENA countries that are predominantly Muslim, as “Middle Eastern” was the term our subject population reported most commonly using and understanding. In addition, in line with APA style and other inputs (e.g., Appiah 2020) we capitalize “White” throughout when referring to the racial group.

3 As targets engaged in the interview, it could be argued that they were presumably “thinking” during the entire interview and thus should have reported a thought after each question. However, the pattern from now dozens of studies using Ickes’ paradigm (for reviews, see Hodges et al. 2015; Ickes and Hodges 2013) suggests that instead of providing a continuous stream of consciousness, targets instead report discrete mental events, e.g., being surprised by something, forming a judgment about something, wondering about something. Targets’ responses often include a mix of thoughts and feelings, e.g., “I was thinking about how happy she made me feel.”

4 In hindsight, we regretted not limiting participation in this phase of this study to non-MENA subjects, and we would definitely limit participation in this way in any future studies on this topic. One participant of MENA ethnicity reported being very offended at being asked about stereotypes of Middle Eastern men, and described the experience as consistent with other prejudice they had experienced in university. Because data collection was conducted online for this phase of this study, the participant’s response was not seen until some time after it was submitted, at which point the participant (whose identity details were largely unknown, due to the online nature of the data collection and the mechanics of the subject pool) was emailed an offer to discuss this study further (the offer was declined). The incident was reported to the university’s office of Human Subjects Compliance.

5 We initially included random slopes for all of the predictors. However, the model with random slopes (as well as random intercepts) did not converge given the small of number of observations for thoughts at Level 1. Thus, we removed the random slopes completely to avoid convergence issues.

6 In their study of stereotypes and empathic accuracy, Lewis et al. (2012) found that women perceivers were more accurate than men perceivers at inferring new mother targets’ thoughts. However, they attributed this difference at least in part to the gendered topic (new motherhood).

## Appendix A. Interview Questions

1. How would you describe yourself?
2. How would you describe the country where you grew up?
3. What is one of your best memories from childhood?
4. What is your favorite food?
5. What did you think about the United States before coming here?
6. What’s your impression of American culture since you’ve been in the United States?
7. What differences, if any, have you noticed about the role women have in American society versus in Middle Eastern society?
8. What role do you think women should have in society?
9. What do you think people should value the most in life?
10. What role does religion play in your life?
11. Are you a part of a certain religious group?
12. What do you think about conflicts that occur between religious groups?
13. What do you think about dating culture in the United States?
14. What types of activities do you do with your friends?
15. If you could change something in the world, what would it be?
16. What’s something that you think other people would be surprised to know about you?

## Appendix B. Stereotype Themes

Describes himself as religious/moral/traditional
Describes himself as bold, confident, strong, or powerful
Describes himself as a family man or honorable
Describes himself as just like anyone else, as normal
Describes himself as smart or intelligent
Describes himself as hard working
Describes himself as good or a kind man
Describes himself as successful or as a businessman
Describes the country he grew up in as poor, rural, or un-modernized
Describes the country he grew up in as violent, war ridden or in conflict
Describes the country he grew up in as hot, dry, or desert-like
Describes the country he grew up in as religious
Describes the country he grew up in as having strict rules or as traditional
Describes the country he grew up in as good or having positive traits
Describes best memories from childhood as family time
Describes best memories from childhood as playing with friends or playing sports
Describes best memories from childhood as religious activities
Favorite food is Middle Eastern food
Favorite food is hummus
Favorite food is lamb
Favorite food is rice
Favorite food is curry
Favorite food is meat or steak
Favorite food is falafel
Favorite food is shawarma
Favorite food is spicy and/or flavorful
Thoughts about U.S. before coming here included opportunity and/or freedom
Thoughts about U.S. before coming here were very negative, or included hating Americans or disagreeing with their views
Thoughts about U.S. before coming here included viewing Americans as hateful, unaccepting, or judgmental
Thoughts about U.S. before coming here included viewing Americans as rich, living in luxury, entitled, or arrogant
Impressions of American culture include viewing it as judgmental or racist
Impressions of American culture include viewing it as immoral or immodest
Impressions of American culture include viewing it as free or open
Impressions of American culture include dislike for or negative opinion of it
Impressions of American culture include perceiving large differences between America and Middle East
Impressions of American culture include perceiving Americans as spoiled, entitled, or lazy
Impressions of American culture include perceiving Americans as having negative opinions of Middle Easterners
Impressions of American culture include perceiving Americans as fat or unhealthy
Perceives American women as having more freedom, rights, opportunities, or equality
Perceives American women as immodest or having too much power and freedom
Perceives women in the Middle East as being treated as caretakers, as modest, as treated as unequally to men, as having less opportunity or as subservient to men
Believes the role women should have in society is as mother, caretaker, or wife
Believes the role women should have in society is lesser role than or role subservient to men
Believes people should value religion most in life
Believes people should value family most in life
Religion plays very important or large role in his life
Being religiously active plays very important or large role in his life
Religion is important to him even if he is not personally religious
Is a member of a Muslim religious group
Is a member of an unspecified religious group
He is assumed to be religious or Muslim by other people, and/or other people misunderstand his religion
He thinks conflict that occurs between religious groups is unnecessary or too harmful and/or that people should strive for peace (he disapproves of religious conflict)
He thinks conflict that occurs between religious groups reflects the importance of religion, reflects that there is one true religion or reflects that these conflicts are sometimes necessary or justified
He thinks dating culture in the United States is wrong or immoral
He thinks dating culture in the United States is too casual (thinks dating is not taken seriously enough)
He thinks dating culture in the United States is open, free, and casual, without judging it as negative
Likes to play sports, games, or soccer with friends
Likes to eat, drink, or talk with friends
Likes to smoke a hookah or smoke with friends
Likes to do religious activities with friends
Likes to go to clubs and bars with friends
